# Development of the Therapeutic Alliance and its Association With Internet-Based Mindfulness-Based Cognitive Therapy for Distressed Cancer Patients: Secondary Analysis of a Multicenter Randomized Controlled Trial

**DOI:** 10.2196/14065

**Published:** 2019-10-18

**Authors:** Else Bisseling, Linda Cillessen, Philip Spinhoven, Melanie Schellekens, Félix Compen, Marije van der Lee, Anne Speckens

**Affiliations:** 1 Radboudumc for Mindfulness Department of Psychiatry Radboud University Medical Center Nijmegen Netherlands; 2 Institute of Psychology Leiden University Leiden Netherlands; 3 Department of Psychiatry Leiden University Medical Center Leiden Netherlands; 4 Helen Dowling Institute Centre for Psycho-Oncology Scientific Research Department Bilthoven Netherlands

**Keywords:** therapeutic alliance, telemedicine, mindfulness, cancer, patient dropouts

## Abstract

**Background:**

Mindfulness-based cognitive therapy (MBCT) is an evidence-based group-based psychological treatment in oncology, resulting in reduction of depressive and anxiety symptoms. Internet-based MBCT (eMBCT) has been found to be an effective alternative for MBCT. The therapeutic alliance (the bond between therapist and patient,) is known to have a significant impact on psychological treatment outcomes, including MBCT. A primary concern in the practice of eMBCT is whether a good therapeutic alliance can develop. Although evidence for the beneficial effect of therapist assistance on treatment outcome in internet-based interventions (IBIs) is accumulating, it is still unclear whether the therapeutic alliance is related to outcome in IBIs.

**Objective:**

This study aimed to (1) explore whether early therapeutic alliance predicts treatment dropout in MBCT or eMBCT, (2) compare the development of the therapeutic alliance during eMBCT and MBCT, and (3) examine whether early therapeutic alliance is a predictor of the reduction of psychological distress and the increase of mental well-being at posttreatment in both conditions.

**Methods:**

This study was part of a multicenter randomized controlled trial (n=245) on the effectiveness of MBCT or eMBCT for distressed cancer patients. The therapeutic alliance was measured at the start of week 2 (ie, early therapeutic alliance), week 5, and week 9. Outcome measures were psychological distress, measured with the Hospital Anxiety and Depression Scale, and mental well-being, measured with the Mental Health Continuum-Short Form.

**Results:**

The strength of early therapeutic alliance did not predict treatment dropout in MBCT or eMBCT (B=−.39; *P*=.21). Therapeutic alliance increased over time in both conditions (*F*_2,90_=16.46; Wilks λ=0.732; *P*<.001). This increase did not differ between eMBCT and MBCT (*F*_1,91_=0.114; *P*=.74). Therapeutic alliance at week 2 predicted a decrease in psychological distress (*B*=−.12; *t*_114_=−2.656; *P*=.01) and an increase in mental well-being (*B*=.23; *t*_113_=2.651; *P*=.01) at posttreatment. The relationship with reduction of psychological distress differed between treatments: a weaker early therapeutic alliance predicted higher psychological distress at posttreatment in MBCT but not in eMBCT (*B*=.22; *t*_113_=2.261; *P*=.03).

**Conclusions:**

A therapeutic alliance can develop in both eMBCT and MBCT. Findings revealed that the strength of early alliance did not predict treatment dropout. Furthermore, the level of therapeutic alliance predicted reduced psychological distress and increased mental well-being at posttreatment in both conditions. Interestingly, the strength of therapeutic alliance appeared to be more related to treatment outcome in group-based MBCT than in eMBCT.

**Trial Registration:**

ClinicalTrials.gov NCT02138513; https://clinicaltrials.gov/ct2/show/NCT02138513

## Introduction

### Background

From 2025 onward, 20 million people worldwide will be diagnosed with cancer each year [1]. Approximately one-third of all cancer patients suffer from psychological distress [2], resulting in long-lasting reduced quality of life, decreased compliance with medical care, and prolonged duration of hospital stay [3,4]. Mindfulness-based interventions (MBIs) such as mindfulness-based cognitive therapy (MBCT) [5] are viable psychological treatment options for cancer patients. Mindfulness is defined as “Paying attention; on purpose, in the present moment and non-judgmentally” [6]. MBIs are effective in reducing psychological distress in cancer patients [7,8]. However, as MBIs typically require in-person attendance at a series of classes over several weeks, many cancer patients experience barriers to participate due to illness, side effects of anticancer treatment, fatigue, limited mobility, or transport options [9].

Internet-based MBCT (eMBCT), overcoming many of these barriers, was found to be as effective as face-to-face (f2f) group-based MBCT in reducing psychological distress and in improving positive mental health and quality of life in a multicenter randomized controlled trial (RCT) in cancer patients [10]. Although treatment dropout was higher in eMBCT than in MBCT, this did not influence treatment efficacy. In the long term, the reduction of psychological distress was significantly higher in eMBCT than in MBCT [11].

The effectiveness of a psychological intervention depends on therapist skills and the strength of the established therapeutic relationship or the therapeutic alliance [12,13]. Therapeutic alliance is defined as the collaborative and affective bond between the therapist and the patient [14,15]. A good alliance means that the patient and therapist are working well toward the goals of the therapy [16]. The quality of the therapeutic alliance can be attributed to the change in outcome of psychological interventions [17,18]. Alliance measures can assess this working relationship [16]. The therapeutic alliance is often assessed with the Working Alliance Inventory (WAI) [19], measuring the degree of mutual trust between the client and therapist, their agreement on treatment goals, and their agreement on how to reach these goals. Alliance is commonly measured early, middle, and late in therapy. Early alliance strength does seem to be associated with outcome [16]. The significant impact of therapeutic alliance on f2f psychotherapy outcomes has been demonstrated in several meta-analyses [18,20]. It was recently found that therapeutic alliance also predicts the outcome of MBCT in cancer patients [21].

A systematic review of the therapeutic alliance in internet-based interventions (IBIs) indicated the development of therapeutic alliance to be equivalent to its development in f2f therapy [22]. In addition, Reynolds found that clients (n=30) and therapists (n=30) rated their session impact and alliances in Web-based text psychotherapy as strong as previously found in f2f psychotherapy [23]. With regard to the association between therapeutic alliance and outcome, findings are mixed. Earlier research found an association between therapeutic alliance and outcome in internet-based cognitive behavioral therapy (CBT) for depression and symptoms of posttraumatic stress [24,25] and in blended CBT for depression [26]. In a sample of cancer survivors (n=46), it was found that a strong therapeutic alliance predicted depressive symptom reduction in telephone-assisted CBT [27]. However, in other studies, no significant relationship between treatment outcome and the therapeutic alliance was found [28,29]. As far as we know, no studies have been conducted on the association of therapeutic alliance and outcome in eMBCT, although online mindfulness interventions are increasingly used [30]. To date, findings in internet-based formats are uncertain, and the relationship between therapeutic alliance and outcome of internet-based psychological interventions remains understudied [31]. The main aim of this study was to contribute to the ongoing debate in psychotherapy research on the relative importance of the therapeutic relationship on treatment outcome in (internet-based) psychological interventions. Gaining knowledge about whether therapeutic aspects, such as therapeutic alliance, influence outcome in eMBCT might improve therapeutic strategies and adequate implementation strategies.

### Objectives

The objectives of this study were to (1) explore whether early therapeutic alliance predicts treatment dropout in MBCT or eMBCT, (2) compare the development of the therapeutic alliance during eMBCT and MBCT, and (3) examine whether early therapeutic alliance is a predictor of the reduction of psychological distress and the increase of mental well-being at posttreatment in both conditions.

## Methods

### Design

This study was part of a large multicenter RCT (n=245) on the effectiveness of MBCT and eMBCT versus treatment as usual (TAU) for distressed cancer patients. The study was registered on Clinicaltrials.gov (NCT02138513) shortly after the start of recruitment, reported following CONSORT guidelines [32], and a protocol paper was published in advance [10,33]. Participants were randomized to MBCT, eMBCT, or TAU. After 3 months, patients in TAU were randomly allocated to either MBCT or eMBCT.

This study involved data from all patients randomized to MBCT or eMBCT, including those patients who were randomized to MBCT or eMBCT after they had completed the 3-month TAU period. The local ethics committee approved this study (CMO Arnhem Nijmegen 2013/542). All participants provided written informed consent before study enrolment.

### Study Population and Procedure

Patients were recruited in specialized mental health care institutes for psycho-oncology via social media, patient associations, and advertorials in local newspapers in the Netherlands. Patients who were interested in participation could enroll themselves at the study website for a screening assessment with the Hospital Anxiety and Depression Scale (HADS). Patients with a score of 11 or greater on the HADS were contacted by telephone by one of the researchers to assess eligibility. Inclusion criteria were as follows: (1) having any cancer diagnosis, (2) experiencing at least mild psychological distress, (3) computer literacy and access to the internet, (4) good command of the Dutch language, and (5) willingness to participate in either online or f2f group–based MBCT. Exclusion criteria were as follows: (1) severe psychiatric morbidity such as suicidal ideation or psychosis, (2) change in psychotropic medication within 3 months of baseline, and (3) current or previous participation in MBCT or mindfulness-based stress reduction. Before randomization, patients completed the baseline assessment.

### Intervention and Therapists

MBIs, such as MBCT [5], are innovative and effective psychological treatment options and have increasingly been applied in somatic health care, including oncology. Mindfulness is defined as intentionally paying attention to present-moment experiences in an accepting and nonjudgmental way [34]. They were originally developed for patients with chronic somatic conditions to help them cope more effectively with their condition, by increasing the capacity to focus on present-moment experiences, even in the presence of internal discomfort, and furthermore to prevent relapse in patients with recurrent major depressive disorders [5]. More recently, it has been applied in other conditions [35]. A 2012 meta-analysis of 9 RCTs (n=955) of MBIs in cancer patients found significant improvements in depressive and anxiety symptoms [7]. Since then, a number of RCTs have replicated the reduction of distress following MBIs [36-40].

#### Group-Based Mindfulness-Based Cognitive Therapy

Patients randomized to group-based MBCT received the intervention according to the MBCT protocol of Segal et al [5]. The MBCT protocol was tailored to cancer patients by including cancer-related psychoeducation and adapted movement exercises. The group-based MBCT consisted of 8 weekly 2.5-hour group sessions, one 6-hour silent day, and daily home practice assignments guided by audio files. The sessions consisted of mindfulness practices, sharing experiences, and didactic teachings. Each participant received a folder with information on each session and a CD containing the audio files. The group-based MBCT was provided at the Radboud University Medical Center in Nijmegen, the Jeroen Bosch Hospital in 's-Hertogenbosch, and 4 mental health institutes specialized in psycho-oncology (Helen Dowling Institute in Bilthoven, Ingeborg Douwes Centrum in Amsterdam, de Vruchtenburg in Rotterdam/Leiden, and Het Behouden Huys in Haren).

#### Internet-Based Mindfulness-Based Cognitive Therapy

The eMBCT followed exactly the same protocol as the group-based MBCT. The eMBCT was mainly text based and included asynchronous written interaction with a therapist over email, similar to the study by Bruggeman-Everts et al [41]. Patients were given access to a website divided into a workspace containing 9 sessions (8 weeks+1 full-day silent retreat) and a secured inbox. The silent day consists of a 6-hour program of practicing mindfulness in silence, with various exercises, and a lunch and tea breaks in silence. The therapist initiated the eMBCT by sending a welcome message to the patient, personalized with a photo or brief description of oneself as introduction. When patients logged in, they were presented with the overview of all information and assignments due for that week. Each session contained an introductory text; daily formal (eg, sitting meditation) and informal exercises (eg, awareness of everyday activities); and other home practice, with guided audio files and accompanying diaries. They were shown (fictional) patients’ descriptions to emphasize common experiences and clarify the use of the logfiles. The therapist provided written feedback on the completed logfiles via the secured inbox on a prearranged day of the week. Patients were given written instructions after week 5 to prepare for their silent day at home. In the week of the silent day, patients were provided with a program similar to the MBCT silent day. Having completed 4 or more sessions of MBCT was defined as a minimum adequate dose in both interventions [42].

#### Therapists

A total of 14 therapists participated: 7 taught both interventions, 2 taught only MBCT, and 5 taught only eMBCT. All therapists fulfilled the advanced criteria of the Association of Mindfulness-Based Teachers in the Netherlands and Flanders, which are in concordance with the UK Mindfulness-Based Teacher Trainer Network Good Practice Guidelines [43]. All therapists had previous experience in working with oncology patients and received a 2-day training workshop in the MBCT study protocol at the start of the study. Moreover, 2 additional day-long supervision meetings were organized during the intervention phase of 1 year and 6 months. Therapists without previous eMBCT experience were provided with guidelines and were supervised by more experienced eMBCT therapists.

### Measures

#### Therapeutic Alliance

Therapeutic alliance was measured with a translated and shortened form of the *WAI* [19], which was administered at the start of week 2, week 5, and week 9. The WAI consists of 3 subscales assessing (1) how closely client and therapist agree on and are mutually engaged in the goals of treatment; (2) how closely client and therapist agree on how to reach the treatment goals; and (3) the degree of mutual trust, acceptance, and confidence between the client and therapist. Items were scored on a 5-point scale ranging from rarely (0) to always (5) [44,45]. The 12-item inventory was validated in a Dutch-speaking sample, showing an internal consistency of greater than 0.80 for all separate subscales and 0.87 for the total scale [46]. The scale was used before in oncology patients following eMBCT [47]. Internal consistency of the total scale of the version used in this study was high (Cronbach alpha=.93).

#### Psychological Distress

Psychological distress was measured with the 14-item *HADS* developed to measure depression and anxiety in general medical populations [32]. Items were scored on a 4-point scale ranging from rarely (0) to almost always (4). It has been validated in medical populations, including cancer patients [48], and has adequate psychometric properties to detect distress and to screen for psychiatric disorders in cancer patients [49,50]. Internal consistency of the total scale in the present sample was high (Cronbach alpha=.87). Patients completed the HADS before and after the intervention.

#### Mental Well-Being

Mental well-being was measured by the *Mental Health Continuum-Short Form* (MHC-SF) [51], which comprises 14 items, representing various feelings of well-being in the past month rated on a 6-point Likert scale from never (0), once or twice a week (1), about once a week (2), 2 or 3 times a week (3), almost every day (4), and every day (5). The MHC-SF contains 3 subscales: emotional, psychological, and social well-being. The short form of the MHC-SF has shown excellent internal consistency (>0.80). The test-retest reliability of the MHC-SF over 3 successive 3-month periods was 0.68, and the 9-month test-retest in a Dutch sample was 0.65 [52]. Internal consistency of the total scale in the sample used in this study was high (Cronbach alpha=.97). Patients completed the MHC-SF before and after the intervention.

### Statistical Analyses

All analyses were conducted in SPSS version 25.0 (IBM Corp, 2017). Differences between conditions and between completers and noncompleters (<4 sessions attended) in demographic and clinical variables were tested with chi-square tests and *t* tests. To test whether strength of alliance predicted dropout of eMBCT and MBCT, binary logistic regression analyses were used.

For further analyses, we only included patients who completed MBCT or eMBCT (n=163). To test the development of the therapeutic alliance during eMBCT and MBCT, repeated-measures analyses of variance were used. To examine whether therapeutic alliance predicted outcome and whether this was different in the 2 conditions, separate hierarchical linear regression models were conducted. The dependent variable was posttreatment psychological distress (HADS) or mental well-being (MHC-SF). In the first step, independent variables were the baseline level of psychological distress or mental well-being and condition (MBCT/eMBCT). In the second step, therapeutic alliance at week 2 was included as predictor. In the third step, the interaction between condition and therapeutic alliance was added to test moderation.

## Results

### Participants

**Figure 1:**
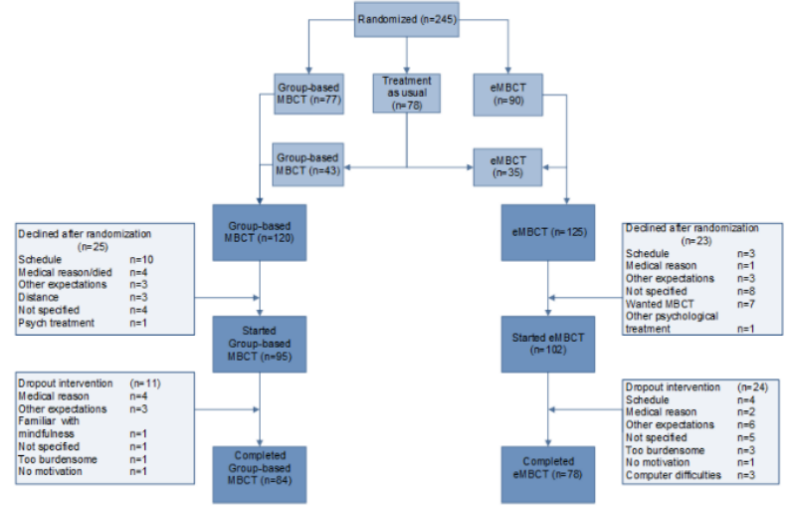
Flowchart of the sample used in the present study. eMBCT: internet-based mindfulness-based cognitive therapy; MBCT: mindfulness-based cognitive therapy.

Of the cancer patients participating in the RCT (n=245), 125 were randomized to eMBCT and 120 were randomized to MBCT. Of all patients (n=125) randomized to eMBCT, 22 (19.2%) declined participation after randomization. Of all patients (n=120) randomized to MBCT, 25 (20.8%) declined participation after randomization (see Figure 1). Participants were mostly female, middle aged, and highly educated. Most patients were diagnosed with breast cancer and were treated with a curative intent (see Table 1).

**Table 1:** Sample characteristics per intervention (mindfulness-based cognitive therapy/internet-based mindfulness-based cognitive therapy).

Characteristics			Mindfulness-based cognitive therapy^a^ (n=120)	Internet-based mindfulness-based cognitive therapy^a^ (n=125)	Chi-square (*df*)	*t* test (*df*)	*P* value
**Sociodemographics**							
	Gender (female), n (%)		101 (84.2)	109 (87.2)	0.5 (1,244)	—^b^	.50
	Relation (yes), n (%)		102 (85.0)	100 (80.0)	1.6 (1,244)	—	.40
	Children, n (%)		79 (65.8)	90 (72.0)	1.1 (1,244)	—	.30
	**Education level, n (%)**						
		Low/middle	35 (29.2)	44 (35.4)	1.02 (1,244)	—	.31
		High	85 (70.8)	81 (64.8)	1.02 (1,244)	—	.31
	Age (years), mean (SD)		51.5 (11.1)	51.8 (10.2)	—	−0.18 (243)	.86
**Clinical**							
	**Cancer diagnosis,** **n (%)**						
		Breast	75 (62.5)	76 (60.8)	0.08 (1,244)	—	.78
		Other	45 (37.5)	49 (39.2)	0.08 (1,244)	—	.78
	**Treatment intent, n (%)**						
		Curative	104 (86.7)	102 (81.6)	1.17 (1,244)	—	.28
		Palliative	16 (13.3)	23 (18.4)	1.17 (1,244)	—	.28
	Current anticancer treatment (yes), n (%)		56 (46.7)	60 (48.0)	0.44 (1,244)	—	.83
	Psychiatric diagnosis (yes), n (%)		44 (36.0)	45 (36.7)	0.012 (1,244)	—	.91
	Time since diagnosis, mean (SD)		3.5 (5.0)	3.4 (4.4)		0.21 (243)	.84
	Psychological distress, Hospital Anxiety and Depression Scale, mean (SD)		18.2 (6.7)	16.9 (6.9)	1.49 (1,244)	—	.14
	Mental well-being, Mental Health Continuum-Short Form, mean (SD)		35.0 (12.8)	37.4 (13.6)	−1.46 (1,244)	—	.15

^a^Both categories include the patients that were initially randomized to the treatment as usual group.

^b^Not applicable.

### Dropout

Of the patients who started MBCT or eMBCT (n=198), 35 (17.7%) dropped out of the intervention. Dropout rate in eMBCT (n=24, 12.1%) was significantly higher than that in MBCT (n=11, 5.6%; *P*=.03). There were no differences in sociodemographics between participants who completed MBCT or eMBCT and those who did not.

Patients who dropped out after the first sessions did not fill out the WAI questionnaire and, consequently, were not included in the analyses testing whether early therapeutic alliance predicted dropout of eMBCT and MBCT. However, reasons for dropout after session 1 were collected. Main reason was that the intervention was not as expected (n=6, 25.7%). Other reasons for dropout are summarized in Multimedia Appendix 1.

Analyses testing whether early therapeutic alliance predicted dropout of eMBCT and MBCT revealed that there was no significant difference in early therapeutic alliance between participants who dropped out of the intervention (mean 33.3, SD 7.9) and those who completed MBCT or eMBCT (mean 36.6, SD 9.5; *P*=.23). Moreover, this effect was not moderated by treatment condition, as there was no significant interaction between condition and the therapeutic alliance at the start of week 2 in association with dropout (*B*=−.13; *P*=.08).

### Development of Therapeutic Alliance

**Figure 2:**
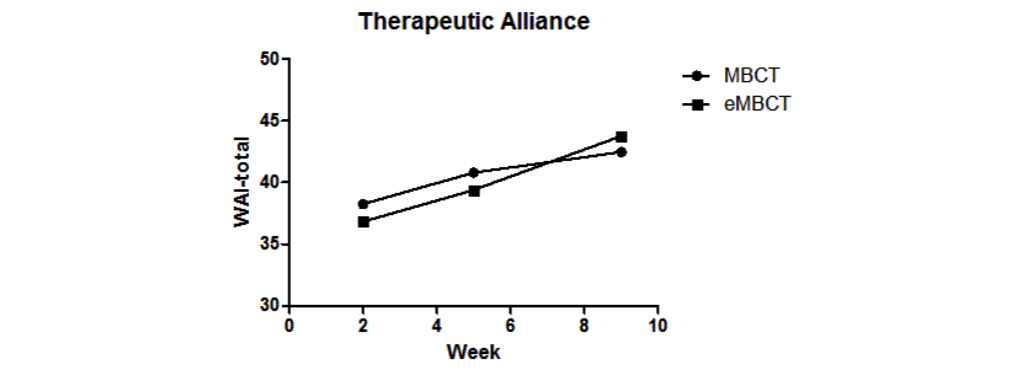
Mean level of therapeutic alliance throughout mindfulness-based cognitive therapy and internet-based mindfulness-based cognitive therapy. eMBCT: internet-based mindfulness-based cognitive therapy; MBCT: mindfulness-based cognitive therapy.

As shown in Figure 2, level of therapeutic alliance increased significantly over time (*F*_2,90_=16.46; Wilks ʎ=0.732; *P*<.001) and did not differ significantly between conditions (*P*=.74). There was no significant interaction between condition and time (*F*_2,90_=1.636; Wilks ʎ=0.965; *P*=.20), implying that the development of therapeutic alliance did not differ between eMBCT and MBCT either.

### Association of Therapeutic Alliance With Treatment Outcome

As you can see in Tables 2 and 3, early therapeutic alliance predicted both reduction of psychological distress (*B*=−.12; t114=−2.656; *P*=.01) and increase of mental well-being (*B*=.23; t113=2.651; *P*=.01) at posttreatment. Adding the interaction term between early therapeutic alliance and condition resulted in a significant increase in explained variance (∆*R*^2^=0.031; *F*_1,114_=6.622; *P=*.01). The relationship between early therapeutic alliance and psychological distress at posttreatment was moderated by condition (*B*=.22; *t*_113_=2.245; *P*=.03).

Examination of the interaction plot showed that participants of the group-based MBCT with a weaker early therapeutic alliance showed a significant higher level of psychological distress at posttreatment than those of eMBCT (*P*=.004; see also Multimedia Appendix 2).

**Table 2:** Relationship between therapeutic alliance and psychological distress: regression analyses. Italicized values indicate significance.

Variables	Regression analyses of psychological distress			Predictor		
	*F* value (*df*)	*P* value	Adjusted *R^2^*	Beta	*t* test	*P* value
Psychological distress baseline	42.72 (2,115)	*<.001*	0.416	.58	8.69 (113)	*<.001*
Condition	42.72 (2,115)	*<.001*	0.416	−2.3	−2.36 (113)	*.01*
Therapeutic alliance week 2^a^	32.08 (3,114)	*<.001*	0.443	−.12	−2.57 (113)	*.01*
Therapeutic alliance week 2×condition^a^	26.17 (4,113)	*<.001*	0.463	.22	2.245 (113)	*.03*

^a^Corrected for baseline psychological distress and the condition, and the predictor×intervention interaction and psychological distress at posttreatment with separate hierarchical linear regressions (n=163).

**Table 3:** Relationship between therapeutic alliance and mental well-being: regression analyses. Italicized values indicate significance.

Variables	Regression analyses of mental well-being			Predictor		
	*F* value (df)	*P* value	Adjusted *R*^*2*^	Beta	*t* test	*P* value
Mental well-being baseline	65.26 (2,115)	<.001	0.523	.76	10.84 (113)	*<.001*
Condition	65.26 (2,115)	<.001	0.523	1.9	1.16 (113)	.24
Therapeutic alliance week 2^a^	48.15 (3,114)	<.001	0.547	.24	2.66 (113)	*.01*
Therapeutic alliance week 2×condition^a^	36.07 (4,113)	<.001	0.545	−.12	−.697 (113)	.49

^a^Corrected for baseline mental well-being and the condition, and the predictor×intervention interaction and mental well-being at posttreatment with separate hierarchical linear regressions (n=163).

## Discussion

### Principal Findings

The aims of this study were to (1) explore whether the strength of early therapeutic alliance predicts treatment dropout in MBCT or eMBCT, (2) compare the development of the therapeutic alliance during eMBCT and MBCT, and (3) examine whether early therapeutic alliance is a predictor of the reduction of psychological distress and the increase of mental well-being at posttreatment in both conditions. Results showed that early alliance did not predict dropout and that the development of the therapeutic alliance did not differ between eMBCT and MBCT. In addition, early therapeutic alliance predicted the reduction of psychological distress and improvement of mental well-being at posttreatment. Moreover, we found that the association between a weaker therapeutic alliance and smaller reduction of psychological distress was stronger in group-based MBCT than in eMBCT.

In our sample, dropout rates in eMBCT were somewhat higher than those in MBCT. This is in line with previous research findings that dropout rates in eMBCT are relatively high [10,53]. We could not confirm previous findings that a strong early alliance prevents patients from dropping out of psychological treatment [54]. In this respect, we would like to stress that our findings should be interpreted with caution. We were unable to include data from patients who dropped out after the first session because early therapeutic alliance was measured at the start of the second session. The main reason for dropping out after the first session was “other expectations.” This finding stresses the importance of clearly explaining the treatment rationale and developing a shared understanding with patients at the start of MBCT or eMBCT. In our study, the therapist initiated the contact in eMBCT by sending a welcome message to the patient. As it was previously found in a qualitative study that in eMBCT asynchronicity of therapist contact/feedback is experienced by some patients as uncomfortable [9], adding an (f2f) appointment with the therapist at the start of eMBCT and synchronous therapist assistance in the early treatment phase might be useful to prevent dropout. Moreover, it was previously found that therapists seem concerned with the level of continuity of the training. Missing out on nonverbal information of patients made them unable to spot withdrawal at an early stage [9]. Alliance building may enhance engagement to treatment. So far, quantitative studies concerning the association of therapeutic alliance and adherence to IBIs are missing. Knowledge about alliance and adherence can be important to define the optimal level of therapist support in IBIs [31].

Regarding the association of therapeutic alliance and outcome, our findings are in line with previous (meta-analytic) research findings, showing that the therapeutic alliance is associated with outcome in f2f psychotherapy [18,20], including MBCT [21] and IBIs [24,25]. An explanation for the finding that the strength of the early alliance affects the outcome more in MBCT than in eMBCT could be that in a group-based setting, patients with a weak therapeutic alliance might conceal themselves in the group and might put in less effort to share experiences or practice. This might explain why psychological distress levels of those patients remained high compared with patients who experienced a strong therapeutic alliance. As a consequence, particularly in group-based settings, mindfulness therapists should be aware of their way of bonding with patients. Although we did not investigate this in our study, it has been previously found that for patients who have difficulties in building a relationship with others, which might be reflected in a weak therapeutic alliance, therapists who model effective ways of forming a strong alliance can process the formation of a strong (repaired) alliance [55,56], which in turn may lead to better outcome. Another explanation for this finding could be that in individual eMBCT, one’s self-efficacy and intrinsic motivation are also important for treatment outcome than the experienced therapeutic alliance [11]. This is in line with previous qualitative findings in eMBCT for cancer patients, which revealed that participants stressed the importance of self-discipline and the individual nature as facilitators for eMBCT [9] suggesting that experienced motivation and discipline is also related to outcome.

### Strengths and Limitations

A major strength of this study was that we directly compared the development of therapeutic alliance between patients participating in eMBCT and MBCT. Except for the delivery format, both interventions were similar in content. Moreover, we investigated the relationship between therapeutic alliance and treatment outcome both in terms of symptom reduction and mental well-being. Some limitations, however, need to be mentioned. At first, this study was conducted in a self-selected group of distressed cancer patients. Although this is in line with patients seeking help in clinical practice, this might limit the extent to which the findings of this study generalize to the broader population of cancer patients [57,58]. Second, an inclusion criterium was that participants would agree with randomization to an f2f or online intervention. Our inclusion criterium might have prevented patients that were not interested in online interventions from participating. This might limit the extent to which the present findings generalize to the broader population of cancer patients.

### Research Implications

Further research should investigate the relation between therapeutic alliance and dropout more thoroughly, for instance, by measuring therapeutic alliance after each session. It would also be interesting to focus on examining what therapeutic techniques might be suitable to improve the development of a strong therapeutic alliance, both in eMBCT and MBCT. Further research in eMBCT could examine how therapeutic alliance can develop in different MBCT or eMBCT formats and how this influences treatment outcome. Treatment formats could vary from blended care to intensive synchronous individual therapist-assisted eMBCT to stand-alone eMBCT with computerized feedback.

### Clinical Implications

As dropout rates were somewhat higher in eMBCT, efforts should be taken to prevent dropout. It is of interest to shed more light on how to encourage patients to adhere to the treatment. Building a good therapeutic alliance is of clinical importance in MBCT or eMBCT. By measuring therapeutic alliance as a routine outcome after every session, therapists can be encouraged to really tune in to patient’s needs and adjust their therapy or their therapeutic attitude at an early treatment stage, which may benefit treatment outcome in both MBCT and eMBCT.

## Appendix

Multimedia Appendix 1 Dropout rates and reasons for dropout per condition.

Multimedia Appendix 2 The association of early therapeutic alliance and outcome per condition.

## Abbreviations

CBT: cognitive behavioral therapy
eMBCT: internet-based mindfulness-based cognitive therapy
f2f: face-to-face
HADS: Hospital Anxiety and Depression Scale
IBI: internet-based intervention
MBCT: mindfulness-based cognitive therapy
MBI: mindfulness-based intervention
RCT: randomized controlled trial
TAU: treatment as usual
WAI: Working Alliance Inventory
