# Supplementary material for: Development of the Therapeutic Alliance and its Association With Internet-Based Mindfulness-Based Cognitive Therapy for Distressed Cancer Patients: Secondary Analysis of a Multicenter Randomized Controlled Trial
Source: J Med Internet Res. 2019 Oct 18;21(10):e14065. doi: 10.2196/14065 (PMC6827984; doi:10.2196/14065)
Supplement: Multimedia Appendix 1 [file jmir_v21i10e14065_app1.pdf]

Table 2. Dropout rates and reasons for dropout per condition.

Dropout	Reason	*After week 1*	*After week 2*	*After week 3*	*Total*
MBCT	Other expectations	2	1	0	3
	Too burdensome	0	1	0	1
	No reason provided	0	0	1	1
	Schedule	0	0	0	0
	Familiar with mindfulness	1	0	0	1
	No motivation	1	0	0	1
	Medical reason	1	1	2	4
	Computer difficulties	0	0	0	0
		5	3	3	11
eMBCT	Other expectations	4	1	1	6
	Too burdensome	1	1	1	3
	No reason provided	3	1	1	5
	Schedule	2	1	1	4
	Familiar with mindfulness	0	0	0	0
	No motivation	0	0	0	1
	Medical reason	0	1	1	2
	Computer difficulties	2	1	0	3
		13	6	5	24
