# Supplementary figures and images for: Development of the Therapeutic Alliance and its Association With Internet-Based Mindfulness-Based Cognitive Therapy for Distressed Cancer Patients: Secondary Analysis of a Multicenter Randomized Controlled Trial
Source: J Med Internet Res. 2019 Oct 18;21(10):e14065. doi: 10.2196/14065 (PMC6827984; doi:10.2196/14065)

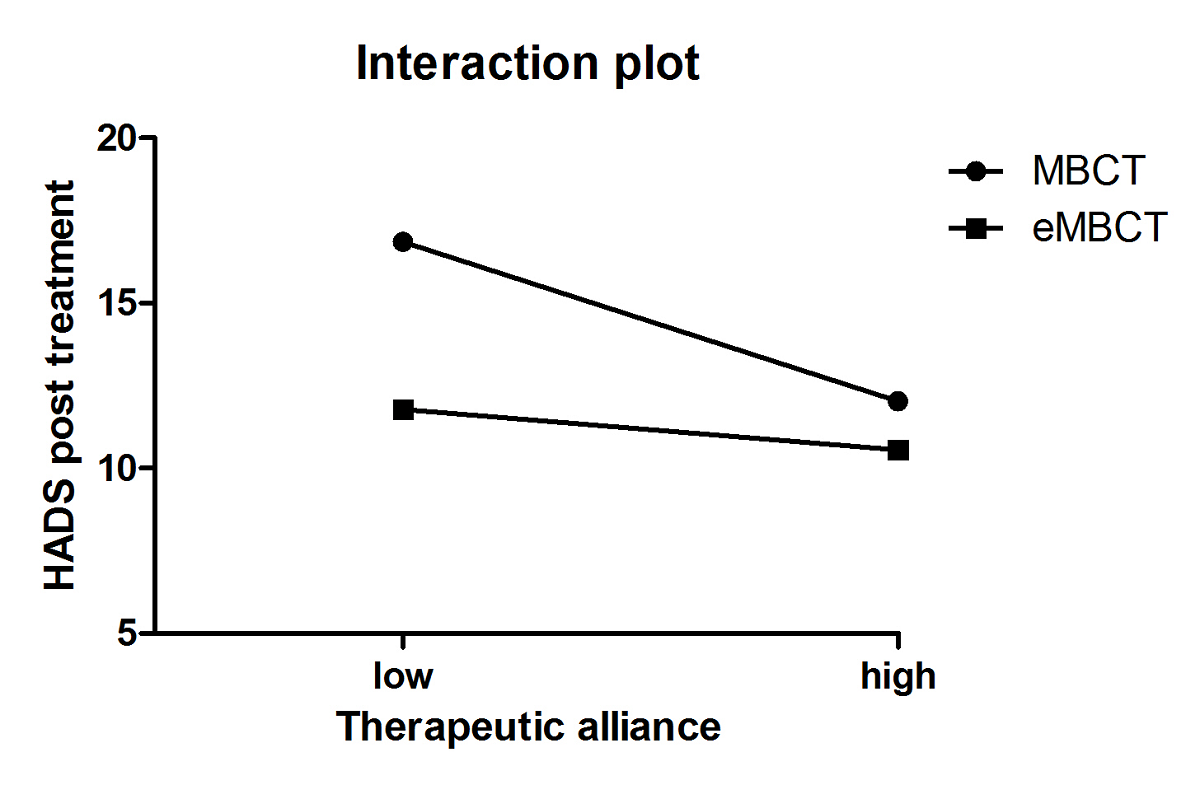

Supplement: Multimedia Appendix 2 [file jmir_v21i10e14065_app2.png]
